# Prevalence of Hepatitis C Infection and Treatment Initiation Following Dried Blood Spot Testing in New South Wales, Australia

**DOI:** 10.1111/liv.70937

**Published:** 2026-10-16

**Authors:** Annabelle Stevens, Anna Conway, Nigel Carrington, Evan B. Cunningham, Alison D. Marshall, Janaki Amin, Phillip Read, Tracey Brown, Rick Varma, Bianca Prain, Philip Cunningham, Gregory J. Dore, Jason Grebely

**Affiliations:** ^1^ The Kirby Institute University of New South Wales Sydney New South Wales Australia; ^2^ NSW Health St Leonards New South Wales Australia; ^3^ Sydney Sexual Health Centre, South Eastern Sydney Local Health District NSW Health Sydney New South Wales Australia; ^4^ Centre for Social Research in Health University of New South Wales Sydney New South Wales Australia; ^5^ Department of Health Sciences, Faculty of Medicine and Health Sciences Macquarie University Sydney New South Wales Australia; ^6^ Sexual Health and Blood Borne Virus Services, South Eastern Sydney Local Health District NSW Health Sydney New South Wales Australia; ^7^ Justice Health and Forensic Mental Health Network NSW Health Sydney New South Wales Australia; ^8^ NSW State Reference Laboratory for HIV St Vincent's Hospital Sydney New South Wales Australia

**Keywords:** dried blood spot, hepatitis C, PWID, testing, treatment

## Abstract

**Background and Aims:**

To achieve hepatitis C (HCV) elimination by 2030, testing and treatment must reach people most at risk. Dried blood spot (DBS) testing offers an alternative to venepuncture, suitable for community and prison settings. This study evaluated HCV infection prevalence and treatment initiation among people receiving DBS testing in New South Wales (NSW), Australia.

**Methods:**

The NSW DBS Pilot was an observational study of people testing for HCV RNA from DBS samples collected across 36 community sites (including drug treatment clinics and needle and syringe programmes) and 21 prisons. Logistic regression identified factors associated with current HCV infection (detectable HCV RNA) and treatment initiation within 6 months.

**Results:**

Between September 2019 and December 2023, 13 940 participants were tested for HCV (25% female, 33% Aboriginal and/or Torres Strait Islander, 36% injected drugs in the last month). Overall, 11% (1310/13940) had current infection, higher in community (12%) than prison (9%), and declining from 14% in 2019–2021 to 9% in 2022–2023. Among those with current infection, treatment initiation within 6 months was higher in prison settings (64%) compared to community (37%). In community settings, Aboriginal and/or Torres Strait Islander people had lower odds of treatment initiation (aOR 0.69, 95% CI: 0.51–0.92). In prison, treatment declined in 2022–2023 compared with 2019–2021 (aOR 0.64, 95% CI: 0.43–0.94).

**Conclusions:**

The NSW DBS Pilot provided broad access to HCV testing, but treatment initiation remained suboptimal, particularly in community settings. Declining prevalence indicates progress towards elimination; however, further efforts are needed to close remaining gaps along the care cascade.

## Key Points

- Dried blood spot testing was implemented across community and prison settings in New South Wales, Australia.
- Hepatitis C infection prevalence declined over time, indicating progress towards elimination.
- Treatment uptake was higher in prisons than in community settings.
- Dried blood spot testing expands access to diagnosis, but treatment gaps remain.

## Summary

Dried blood spot testing has increased access to hepatitis C testing for people at higher risk of infection in community and prison settings across New South Wales, Australia. Although fewer people were living with hepatitis C over time, many did not start treatment, particularly in community settings. Improving access to treatment after testing will be essential to achieve hepatitis C elimination.

**Abbreviations**
AIC: Akaike information criterion
aOR: adjusted odds ratio
BIC: Bayesian information criterion
CI: confidence interval
DAAs: direct‐acting antivirals
DBS: dried blood spot
HCV: hepatitis C virus
HIV: human immunodeficiency virus
IQR: interquartile range
MSM: gay and other men who have sex with men
NSW: New South Wales
RNA: ribonucleic acid
SD: standard deviation
WHO: World Health Organisation

## 1 Introduction

In 2016, the World Health Organisation set a target to eliminate hepatitis C (HCV) as a public health concern by 2030, aiming for a 90% reduction in new infections and a 65% reduction in HCV‐related mortality [1]. Progress has been facilitated by the advent of direct‐acting antivirals (DAAs) for the treatment of HCV that offer cure rates of over > 95% [2]. Many people at risk of HCV infection, including people who inject drugs, may be left behind by elimination efforts due to service‐ and patient‐level barriers, including poor venous access and stigma and discrimination in healthcare settings [3, 4, 5, 6]. To overcome these barriers, elimination of HCV requires tailored testing and treatment strategies for people at risk of infection [7, 8].

Dried blood spot (DBS) testing provides a simple alternative to venepuncture as it can be collected via finger‐stick and remains stable at room temperature, enabling sampling to be conducted in non‐clinical environments such as outreach, community or prison settings [9, 10] or via peer‐led screening to reach people who might not otherwise engage with traditional healthcare services [11]. The diagnostic performance of DBS testing for HCV infection is well established, with studies demonstrating high sensitivity and specificity for detecting HCV RNA [12]. In New South Wales (NSW), Australia, early evaluations demonstrated that DBS was feasible and acceptable for HCV diagnosis [13] and had the potential to increase access to testing among populations at risk of infection [14]. However, less is known about the impact of DBS when implemented at scale within routine health services. Most previous evaluations were conducted prior to 2020 and involved relatively small sample sizes or limited implementation settings [15, 16, 17, 18, 19]. Incomplete data on the characteristics of people receiving DBS testing, the burden of current HCV infection identified, and linkage to antiviral treatment following diagnosis limit understanding of the role of DBS in HCV elimination strategies across diverse service settings.

Since 2017, the NSW DBS Pilot has implemented DBS testing across community and correctional settings throughout NSW. Operating across 36 community sites and 21 correctional centres, the programme provides a unique opportunity to evaluate the large‐scale implementation of DBS testing within routine service delivery. This analysis evaluated the prevalence of current HCV infection, treatment initiation, and associated factors among people receiving testing for HCV RNA in the NSW DBS Pilot.

## 2 Methods

### 2.1 Study Design and Population

The NSW DBS Pilot is a statewide observational cohort study evaluating the scale‐up of HCV and HIV testing from DBS sample collection and has been described previously [13, 14]. Participants were recruited via three pathways (online self‐registration for at‐home sample collection, assisted registration at community sites, or assisted registration in prison) for HIV and/or HCV testing. Participants were recruited across NSW, online, at 36 community sites (providing some or all of the following services: drug treatment, needle and syringe provision, sexual health), and at 21 prisons including male and female reception prisons for new inmates, remand centres, and maximum‐security to minimum‐security correctional centres.

The NSW DBS Pilot recruited people who provided informed consent and were aged ≥ 16 years. The NSW DBS Pilot began in November 2016, offering HIV testing. From September 2017, inclusion criteria for HCV testing were identifying as Aboriginal or Torres Strait Islander or having a history of injecting drug use. From June 2019, inclusion criteria were expanded to people born in Asia or Africa and people who had ever been incarcerated. From September 2019, the eligibility criteria were expanded to include people born in any country other than Australia and the data collection tool was updated to identify participants with a history of injecting drugs in the past month. From July 2021, inclusion criteria for HCV testing were further expanded to include people who are or have been clients of community corrections services, people who are using or have used drug and alcohol services or mental health services, and people who are currently or have ever been homeless. Participants who met the inclusion criteria for HCV testing were eligible for HIV testing. People who met the inclusion criteria for HIV testing (gay and other men who have sex with men [MSM], people from sub‐Saharan Africa and Southeast Asia, and people with current/previous sexual partners from sub‐Saharan Africa and Southeast Asia) were not automatically eligible for HCV testing. Due to multiple amendments in study eligibility criteria and the data collection tool, this analysis reports data from people receiving HCV DBS testing as part of assisted registration at community sites or assisted registration in prison between 1 September 2019 and 31 December 2023 and people initiating treatment until 30 June 2024. Prior research has been completed on DBS testing and treatment outcomes in earlier years of the study [13, 14].

All participants provided informed consent via an online Participant Information Sheet and Consent Form or via verbal consent taken by a staff member (verbal consent was available from August 2023). The initial study protocol and all subsequent amendments were approved by St Vincent's Hospital (Sydney) Human Research Ethics Committee (2019/ETH09614 HREC/15/SVH/400), and additional ethics approval was received from the Aboriginal Health and Medical Research Council Human Research Ethics Committee, NSW Corrective Services Ethics Committee, and Local Health District governance.

### 2.2 Procedures

DBS collection kits contained a test card, a lancet, alcohol swabs, band‐aids, cotton balls, a foil envelope, and a reply‐paid envelope. Procedures for DBS sample collection and testing (elution, spot size, punching protocols, validation, testing algorithm) have been described elsewhere [20, 21]. The tests used in the NSW DBS Pilot were Murex HIV‐1.2.0 antibody enzyme‐linked immunosorbent assay (Diasorin, Macquarie Park, Australia) and New Lav‐Blot‐1 (Bio‐Rad, Gladesville, Australia) for HIV testing, and Aptima HCV Quant Dx assay (Hologic, Macquarie Park, Australia) for HCV RNA testing. Once a DBS sample was collected and dried, it was mailed back to the NSW State Reference Laboratory for HIV (St Vincent's Hospital, Sydney, Australia). Depending on the request, the card was tested for HIV and/or HCV. The algorithm for testing was based on current evidence on DBS testing [12, 20, 22]. All reactive (HIV antibody) and detectable (HCV RNA) results required a confirmatory test via venepuncture for diagnosis.

For assisted registration in the community or in prison, participants were supported to complete the online survey and according to their eligibility, a DBS sample for HIV or HCV testing was collected. The sample was returned to the laboratory and participants were informed of their results by SMS, phone, or in person at the participating site based on site procedures. Depending on the site, linkage to care post‐diagnosis was the responsibility of the statewide Sexual Health Infolink or clinical staff at the site level. The Sexual Health Infolink or clinic site coordinators submitted a standardised case report form for each participant with current HCV infection including data on HCV treatment initiation and loss to follow‐up at 6 months. For participants in prison, the case report form was completed by prison health staff, which only reported on treatment initiations in the prison setting.

All participants completed an online survey of baseline data, including demographics, behavioural risk, and HIV and HCV testing history. There was no compensation or financial incentive offered as part of the study, but some sites provided this as part of local initiatives.

A unique identifier based on patient name and date of birth was used to identify people who tested more than once in the study period. When present, the first HCV RNA detectable test result was included; otherwise, only the first result was included. Demographic and behavioural characteristics were reported from the enrolment survey. HCV RNA test results were reported from the laboratory database. Treatment initiation was collected from the standardised case report forms completed by sites and the statewide Sexual Health Infolink. All databases were linked using a medical record number unique to each testing episode.

### 2.3 Study Outcomes

The primary study outcome was current HCV infection (defined as detectable HCV RNA). The secondary study outcome, treatment initiation within 6 months of testing, was defined as HCV treatment prescribed within 6 months of the registration date. Observation time for treatment initiation commenced on the date of NSW DBS Pilot enrolment and ended on the date of HCV treatment initiation. Those services that were unable to undertake confirmatory testing and/or initiate treatment onsite referred participants to other services. Participants who were treated at other services were not included in the treatment initiation outcome for logistic regression analysis, as the date of treatment initiation was not reported.

### 2.4 Exposures

Demographic and behavioural factors hypothesised to be associated with current HCV infection and treatment initiation were determined from the literature [23, 24, 25, 26] and included: (i) age at survey (five categories: 16–24 years, 25–34 years, 35–44 years, 45–54 years and ≥ 55 years), (ii) gender (male, female, other [including nonbinary and transgender]), (iii) Aboriginal or Torres Strait Islander (no, yes), (iv) born outside of Australia (no, yes), (v) recent injecting drug use (never; yes, but not in the previous 30 days; yes, in the previous 30 days; prefer not to say), (vi) incarceration history (no, yes, prefer not to say) and (vii) time period of test (2019–2021 and 2022–2023).

### 2.5 Statistical Analysis

Baseline participant characteristics were summarised using appropriate summary statistics (mean [SD], median [IQR], *n* [%]) and chi‐squared tests to assess for differences. Logistic regression models were used to estimate crude and adjusted odds ratios (aORs) and corresponding 95% confidence intervals to evaluate factors associated with current HCV infection and HCV treatment initiation. Variables were retained in adjusted models according to the Akaike Information Criterion (AIC) and Bayesian Information Criterion (BIC), provided no collinearity was identified. For all analyses, significance was set at an alpha level of *p* ≤ 0.05, and *p*‐values were two‐tailed. All analyses were performed using Stata 18.0 (StataCorp, College Station, TX, USA).

## 3 Results

### 3.1 Sample Characteristics

Overall, 13 940 participants completed DBS HCV testing during the study period (from 1 September 2019 to 31 December 2023), including 8299 (60%) people in community settings and 5641 (40%) people in prison settings (Table 1). The median age of people tested was 40 years (range: 16–90, interquartile range: 31–49), 25% (*n* = 3455) were female, 33% (*n* = 4665) identified as Aboriginal and/or Torres Strait Islander, and 64% (*n* = 8970) had a history of injecting drugs (with 36%, *n* = 5001, indicating injecting in the past month). There were differences among people tested in community settings and prison settings. Compared with those in the community, people tested in prison settings were younger (34 vs. 44 years), more often male (90% vs. 62%), more often Aboriginal and/or Torres Strait Islander people (39% vs. 30%), and less often had a history of injecting drugs (51% vs. 73%).

**TABLE 1:** Baseline characteristics of people completing HCV DBS testing by setting type (*n* = 13 940).

Variables	People tested *N* (%)	People tested in community sites *N* (%)	People tested in prison sites *N* (%)
Total	13 940	8299 (60%)	5641 (40%)
Median age			
< 39	6312 (45%)	2618 (32%)	3694 (65%)
≥ 39	7628 (55%)	5681 (68%)	1947 (35%)
Gender			
Men	10 231 (73%)	5178 (62%)	5053 (90%)
Women	3455 (25%)	2894 (35%)	561 (10%)
Non‐binary/other	254 (2%)	227 (3%)	27 (0%)
Aboriginal and/or torres strait islander			
No	9275 (67%)	5809 (70%)	3466 (61%)
Yes	4665 (33%)	2490 (30%)	2175 (39%)
Born outside of Australia			
No	11 974 (86%)	7078 (85%)	4896 (87%)
Yes	1966 (14%)	1221 (15%)	745 (13%)
History of injecting drugs			
Never	4191 (30%)	1595 (19%)	2596 (46%)
Yes, but not in the previous month	3969 (28%)	2185 (26%)	1784 (32%)
Yes, in the previous month	5001 (36%)	3898 (47%)	1103 (20%)
Prefer not to say	779 (6%)	621 (7%)	158 (3%)
History of incarceration			
No	3165 (23%)	3165 (38%)	0 (0%)
Yes	10 008 (72%)	4367 (53%)	5641 (100%)
Prefer not to say	767 (6%)	767 (9%)	0 (0%)
Time period of test			
2019–2021	5284 (38%)	3019 (36%)	2265 (40%)
2022–2023	8656 (62%)	5280 (64%)	3376 (60%)

### 3.2 Current HCV Infection Among Community Settings

Of those tested for HCV in community settings (*n* = 8299), 12% (*n* = 1020) had current HCV infection. The proportion with current HCV infection declined from 16% in 2019–21 to 10% in 2022–23 (*p* < 0.001). The proportion with current HCV infection was highest in males (13% vs. 10% in women; *p* < 0.001), people born in Australia (13% vs. 9% in people born outside Australia; *p* < 0.001), people who had injected drugs in the past month (18% vs. 12% among people who injected drugs but not in the last 30 days and 1% among people who had never injected drugs; *p* < 0.001), and people with a history of incarceration (16% vs. 7% among people who had no history of incarceration; *p* < 0.001).

Among people who reported injecting drug use in the previous month, the proportion with current HCV infection declined from 30% in 2019–2021 to 21% in 2022–2023 (Figure 1A). Similarly, there was a decrease in the prevalence of current HCV infection among people who had injected previously but not in the previous month, from 17% in 2019–2021 to 9% in 2022–2023.

**FIGURE 1:**
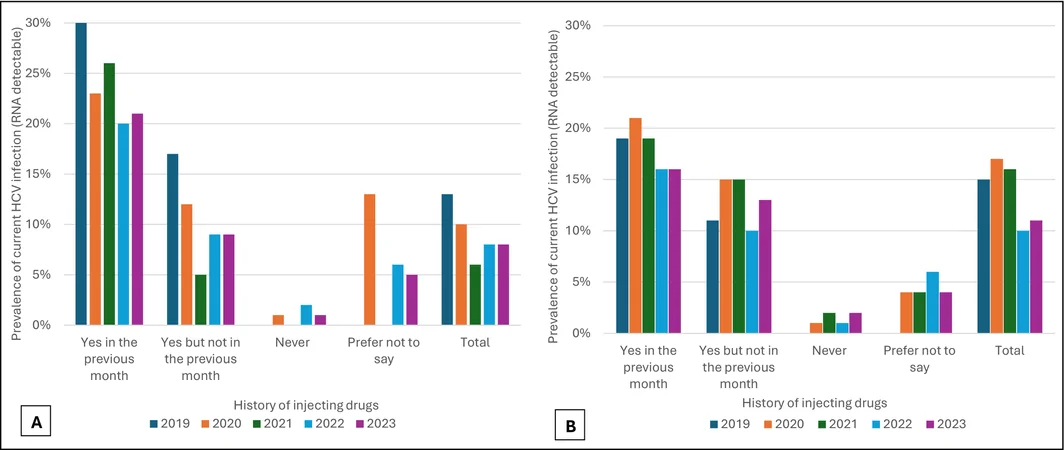
Prevalence of current HCV infection (RNA detectable) by history of injecting drugs and year of test among people completing DBS testing in (A) community settings and (B) prison sites.

In adjusted analyses, current HCV infection was higher among older age groups (35–44 years: aOR 1.50, 95% CI: 1.02–2.20; 45–54 years: aOR 1.66, 95% CI: 1.13–2.43), those with recent injecting drug use (past month: aOR 11.14, 95% CI: 7.21–17.22; not in past month: aOR 7.66, 95% CI: 4.92–11.95), and those with a history of incarceration (aOR 1.65, 95% CI: 1.40–1.94). Being born outside Australia (aOR 0.78, 95% CI: 0.63–0.97) and testing in 2022–2023 (compared with those testing in 2019–2021, aOR 0.74, 95% CI: 0.65–0.85) were associated with lower odds of infection (Table 2).

**TABLE 2:** Factors associated with current HCV infection among HCV DBS tests in community sites (*n* = 1020).

Characteristic	Current HCV infection, *n* (%)	No current HCV infection, *n* (%)	OR (95% CI)	aOR (95% CI)
Total	1020 (12%)	7279 (88%)		
Age				
16–24 years	33 (6%)	523 (94%)	‐ref‐	‐ref‐
25–34 years	150 (11%)	1269 (89%)	1.87 (1.27–2.77)	1.26 (0.85–1.89)
35–44 years	355 (14%)	2178 (86%)	2.58 (1.79–3.74)	1.50 (1.02–2.20)
45–54 years	351 (15%)	2036 (85%)	2.73 (1.89–3.95)	1.66 (1.13–2.43)
55 years and above	131 (9%)	1273 (91%)	1.63 (1.10–2.42)	1.40 (0.93–2.11)
Gender				
Male	698 (13%)	4480 (87%)	‐ref‐	‐ref‐
Female	293 (10%)	2601 (90%)	0.72 (0.63–0.84)	0.90 (0.77–1.05)
Non‐binary/other	29 (13%)	198 (87%)	0.94 (0.63–1.40)	1.52 (0.98–2.36)
Aboriginal or torres strait islander				
No	705 (12%)	5104 (88%)	‐ref‐	
Yes	315 (13%)	2175 (87%)	1.05 (0.91–1.21)	
Born outside of Australia				
No	913 (13%)	6165 (87%)	‐ref‐	‐ref‐
Yes	107 (9%)	1114 (91%)	0.65 (0.53–0.80)	0.78 (0.63–0.97)
Recently injected drugs				
Never	22 (1%)	1573 (99%)	‐ref‐	‐ref‐
Yes, but not in the previous month	271 (12%)	1914 (88%)	10.12 (6.52–15.71)	7.66 (4.92–11.95)
Yes, in the previous month	696 (18%)	3202 (82%)	15.54 (10.12–23.86)	11.14 (7.21–17.22)
Prefer not to say	31 (5%)	590 (95%)	3.76 (2.16–6.54)	3.03 (1.70–5.39)
History of incarceration				
No	232 (7%)	2933 (93%)	‐ref‐	‐ref‐
Yes	706 (16%)	3661 (84%)	2.44 (2.09–2.85)	1.65 (1.40–1.94)
Prefer not to say	82 (11%)	685 (89%)	1.51 (1.16–1.97)	1.37 (1.02–1.83)
Time period of test				
2019–2021	484 (16%)	2535 (84%)	‐ref‐	‐ref‐
2022–2023	536 (10%)	4744 (90%)	0.59 (0.52–0.68)	0.74 (0.65–0.85)

### 3.3 Current HCV Infection Among in Prison Settings

Of those tested in prison (*n* = 5641), 9% (*n* = 490) had current HCV infection. The proportion with current HCV infection declined from 10% in 2019–2021 to 8% in 2022–2023 (*p* < 0.001). The proportion with current HCV infection was highest in males (9% vs. 6% in women; *p* < 0.042), Aboriginal and/or Torres Strait Islander people (12% vs. 6% in non‐Aboriginal and Torres Strait Islander people; *p* < 0.001), people born in Australia (10% vs. 3% in people born outside Australia; *p* < 0.001), and people who had injected drugs in the past month (23% vs. 11% among people who injected drugs but not in the last 30 days and 1% among people who had never injected drugs; *p* < 0.001).

Among those who reported injecting drug use in the previous month, there was a decrease in the proportion with current HCV infection from 19% in 2019 to 16% in 2023 (Figure 1B). Conversely, among people who had injected previously but not in the previous month, the prevalence of current infection did not decline over time, remaining between 13% and 15% across the study period.

In adjusted analyses, there were higher odds of current HCV infection among Aboriginal and/or Torres Strait Islander people (aOR 1.24, 95% CI: 1.01–1.51), and people with a history of injecting drug use (vs never, past month: aOR 19.47, 95% CI: 13.46–28.17; not in past month: aOR 8.35, 95% CI: 5.76–12.10; prefer not to say: aOR 7.35, 95% CI: 3.98–13.58). Older age (vs < 25 years, 24–34 years: aOR 0.73, 95% CI: 0.56–0.94, 35–44 years: aOR 0.70, 95% CI: 0.53–0.93; 45–54 years: aOR 0.67, 95% CI: 0.45–0.99), gender (women: aOR 0.57, 95% CI: 0.39–0.84), and testing in 2022–2023 (aOR 0.74, 95% CI: 0.65–0.85) were associated with lower odds of current HCV infection (Table 3).

**TABLE 3:** Factors associated with current HCV infection among HCV DBS tests in prison sites (*n* = 490).

Characteristic	Current HCV infection, *n* (%)	No current HCV infection, *n* (%)	OR (95% CI)	aOR (95% CI)
Total	490 (9%)	5151 (91%)		
Age				
16–24 years	118 (11%)	910 (89%)	‐ref‐	‐ref‐
25–34 years	195 (9%)	1927 (91%)	0.78 (0.61–0.99)	0.73 (0.56–0.94)
35–44 years	125 (8%)	1407 (92%)	0.69 (0.53–0.89)	0.70 (0.53–0.93)
45–54 years	41 (6%)	600 (94%)	0.53 (0.36–0.76)	0.67 (0.45–0.99)
55 years and above	11 (3%)	307 (97%)	0.28 (0.15–0.52)	0.56 (0.29–1.08)
Gender				
Male	454 (9%)	4599 (91%)	‐ref‐	‐ref‐
Female	33 (6%)	528 (94%)	0.63 (0.44–0.91)	0.57 (0.39–0.84)
Non‐binary/other	3 (11%)	24 (89%)	1.27 (0.38–4.22)	1.10 (0.31–3.91)
Aboriginal or torres strait islander				
No	221 (6%)	3245 (94%)	‐ref‐	‐ref‐
Yes	269 (12%)	1906 (88%)	2.07 (1.72–2.50)	1.24 (1.01–1.51)
Born outside of Australia				
No	468 (10%)	4428 (90%)	‐ref‐	
Yes	22 (3%)	723 (97%)	0.29 (0.19–0.44)	
History of injecting drugs				
Never	35 (1%)	2561 (99%)	‐ref‐	‐ref‐
Yes, but not in the previous month	189 (11%)	1595 (89%)	8.72 (6.05–12.57)	8.35 (5.76–12.10)
Yes, in the previous month	249 (23%)	854 (77%)	21.25 (14.79–30.53)	19.47 (13.46–28.17)
Prefer not to say	17 (11%)	141 (89%)	8.82 (4.82–16.13)	7.35 (3.98–13.58)
Time period of test				
2019–2021	235 (10%)	2030 (90%)	‐ref‐	‐ref‐
2022–2023	255 (8%)	3121 (92%)	0.71 (0.59–0.85)	0.75 (0.61–0.91)

### 3.4 Treatment Initiation in Community Settings

Among those with current HCV infection in community settings, 37% (378/1020) initiated treatment within 6 months of testing and 51% (524/1020) either initiated treatment within 6 months or were referred elsewhere for care. Initiation of treatment within 6 months was higher among men (39% vs. 32% among women; *p* = 0.035) and non‐Aboriginal and/or Torres Strait Islander people (40% vs. 30% among Aboriginal and/or Torres Strait Islander people; *p* = 0.002). After adjustment, Aboriginal and/or Torres Strait Islander identity (aOR 0.69, 95% CI: 0.51–0.92) and time period of test (aOR 0.82, 95% CI: 0.63–1.07) remained the only factors associated with lower odds of HCV treatment initiation within 6 months of testing (Table 4).

**TABLE 4:** Factors associated with treatment initiation within 6 months following DBS HCV RNA detectable result in community sites (*n* = 378).

Characteristic	Treatment initiation within 6 months *N* (% of current infection)	No treatment initiation within 6 months *N* (% of current infection)	OR (95% CI)	aOR (95% CI)
Total	378 (37%)	642 (63%)		
Median age				
16–24 years	11 (33%)	22 (67%)	‐ref‐	
25–34 years	49 (33%)	101 (67%)	0.97 (0.44–2.16)	
35–44 years	124 (35%)	231 (65%)	1.07 (0.50–2.29)	
45–54 years	140 (40%)	211 (60%)	1.32 (0.62–2.80)	
55 years and above	54 (41%)	77 (59%)	1.40 (0.63–3.13)	
Gender				
Male	274 (39%)	424 (61%)	‐ref‐	
Female	94 (32%)	199 (68%)	0.73 (0.55–0.98)	
Non‐binary/other	10 (34%)	19 (66%)	0.82 (0.37–1.78)	
Aboriginal or torres strait islander				
No	283 (40%)	422 (60%)	‐ref‐	‐ref‐
Yes	95 (30%)	220 (70%)	0.64 (0.48–0.85)	0.69 (0.51–0.92)
Born outside of Australia				
No	337 (37%)	576 (63%)	‐ref‐	
Yes	41 (38%)	66 (62%)	1.06 (0.71–1.61)	
History of injecting drugs				
Never	10 (45%)	12 (55%)	‐ref‐	
Yes, but not in the previous month	111 (41%)	160 (59%)	0.83 (0.35–1.99)	
Yes, in the previous month	248 (36%)	448 (64%)	0.66 (0.28–1.55)	
Prefer not to say	9 (29%)	22 (71%)	0.49 (0.16–1.54)	
History of incarceration				
No	91 (39%)	141 (61%)	‐ref‐	
Yes	255 (36%)	451 (64%)	0.87 (0.64–1.18)	
Prefer not to say	32 (39%)	50 (61%)	0.99 (0.59–1.66)	
Time period of test				
2019–2021	189 (39%)	295 (61%)	‐ref‐	‐ref‐
2022–2023	189 (35%)	347 (65%)	0.85 (0.66–1.10)	0.82 (0.63–1.07)

### 3.5 Treatment Initiation in Prison Settings

Among those with current HCV infection in prison settings, 64% (314/490) initiated treatment within 6 months of testing and 74% (365/490) either initiated treatment within 6 months or were referred elsewhere for care. Treatment initiation within 6 months was higher among those tested during 2019–2021 (69% vs. 59% in 2022–2023; *p* = 0.019), and people aged 16–24 years (69% vs. 68% in 25–34 years, 55% in 35–44 years, 66% in 45–54 years and 36% in 55 years and above; *p* = 0.033). In adjusted analyses, testing within 2022–2023 was associated with lower odds of HCV treatment within 6 months (aOR 0.64, 95% CI: 0.43–0.94) (Table 5).

**TABLE 5:** Factors associated with treatment initiation within 6 months following DBS HCV RNA detectable result in prison sites (*n* = 314).

Characteristic	Treatment initiation within 6 months *N* (% of current HCV infection)	No treatment initiation within 6 months *N* (% of current HCV infection)	OR (95% CI)	aOR (95% CI)
Total	314 (64%)	176 (36%)		
Age				
16–24 years	81 (69%)	37 (31%)	‐ref‐	‐ref‐
25–34 years	133 (68%)	62 (32%)	0.98 (0.60–1.60)	1.05 (0.64–1.73)
35–44 years	69 (55%)	56 (45%)	0.56 (0.33–0.95)	0.62 (0.36–1.06)
45–54 years	27 (66%)	14 (34%)	0.88 (0.41–1.87)	0.92 (0.43–1.96)
55 years and above	4 (36%)	7 (64%)	0.26 (0.07–0.95)	0.25 (0.07–0.95)
Gender				
Male	293 (65%)	161 (35%)	‐ref‐	‐ref‐
Female	19 (58%)	14 (42%)	0.75 (0.36–1.53)	0.68 (0.32–1.43)
Non‐binary/other	2 (67%)	1 (33%)	1.10 (0.10–12.21)	1.87 (0.15–22.72)
Aboriginal or torres strait islander				
No	141 (64%)	80 (36%)	‐ref‐	
Yes	173 (64%)	96 (36%)	1.02 (0.71–1.48)	
Born outside of Australia				
No	298 (64%)	170 (36%)	‐ref‐	
Yes	16 (73%)	6 (27%)	1.52 (0.58–3.96)	
History of injecting drugs				
Never	24 (69%)	11 (31%)	‐ref‐	
Yes, but not in the previous month	119 (63%)	70 (37%)	0.79 (0.36–1.70)	
Yes, in the previous month	158 (63%)	91 (37%)	0.79 (0.37–1.69)	
Prefer not to say	13 (76%)	4 (24%)	1.49 (0.39–5.62)	
Time period of test				
2019–2021	163 (69%)	72 (31%)	‐ref‐	‐ref‐
2022–2023	151 (59%)	104 (41%)	0.64 (0.44–0.93)	0.64 (0.43–0.94)

## 4 Discussion

In this study of people at risk of HCV infection receiving DBS testing in NSW, Australia between 2019 and 2023, the prevalence of current HCV infection declined over time in both community and prison settings, reflecting continued progress toward elimination. Prevalence was higher in community sites (12%) than in prison (9%). In adjusted analyses, history of injecting drug use and testing during the 2019–2021 time period were associated with increased odds of current HCV infection in both settings. The proportion of participants with current HCV infection who initiated treatment within 6 months of testing was higher in the prison (64%) compared with the community (37%) although treatment initiation declined in the most recent time period (2022–23). Few demographic or behavioural factors were associated with treatment initiation in either setting, suggesting broadly similar access to care across the characteristics assessed; however, Aboriginal and/or Torres Strait Islander people in the community had significantly lower odds of initiating treatment within 6 months. These findings highlight the need for targeted efforts to improve access and engagement with care among priority populations to support HCV elimination.

The proportion of participants with current HCV infection declined over time in both community and prison settings, likely reflecting the impact of the near‐universal access to DAAs in Australia in 2016 [27] and improved engagement in care among people at risk of infection. This trend aligns with the decreasing prevalence of HCV infection among at‐risk populations reported in recent Australian studies [28, 29] and supports evidence of a population‐wide reduction in current HCV prevalence. Notably, the pattern of HCV prevalence by age differed by setting. In community settings, prevalence increased with age, while in prison settings, HCV prevalence decreased with age. This may reflect higher reinfection rates among younger people in prison or lower treatment initiation in this group. Despite the overall decline, current HCV prevalence remained highest among participants with a history of injecting drugs, particularly those who had recently injected drugs, consistent with existing literature [23, 30, 31, 32]. Given that injecting drug use is the primary mode of transmission of HCV in Australia, reinfection may contribute to ongoing prevalence in this cohort [33]. Notably, even among people with recent injecting drug use, HCV prevalence declined over the study period, likely reflecting the impact of DAA access, improved engagement in care and targeted testing and treatment among at‐risk population groups. These findings emphasise the need for enhanced testing, retreatment and prevention strategies among people who inject drugs, to enhance reductions in HCV prevalence and minimise the risk of reinfection following treatment.

The proportion of participants initiating treatment within 6 months was higher among those tested in prison compared with community sites, reflecting the strength of established models of care within Australian prisons. In NSW, nurse‐led models of care that integrate testing, treatment and follow‐up have been shown to support timely access to treatment [34], likely contributing to higher treatment initiation observed in prisons. The marked difference in treatment initiation between prison (64%) and community settings (37%) highlights an important implementation challenge. While DBS simplifies access to HCV diagnosis, easier diagnosis alone is insufficient to overcome barriers related to treatment initiation. The higher treatment uptake achieved in prison settings than in community settings, where testing is embedded within established treatment pathways, suggests that service delivery models may be as important as testing modality in achieving HCV elimination outcomes. Despite this, treatment uptake in prisons remained suboptimal and was lower in the later time period, suggesting persistent barriers to care. Delays between testing and treatment, as well as possible release from custody before treatment initiation and the need for continuity of care upon release, may contribute to incomplete linkage to care. These delays may be due to structural factors such as limited availability of stocked medications and challenges in arranging follow‐up appointments during incarceration. Point‐of‐care HCV RNA testing may offer a solution to reduce these delays and improve treatment initiation [7, 10, 17, 35, 36, 37]. In contrast, treatment initiation remained lower in the community. As most people with HCV have been treated since the introduction of DAAs [38], tailored strategies are needed to reach the last ‘missing’ who overcome the barriers to care, including homelessness and stigma in healthcare settings [3, 4, 5, 6, 39]. Complex care pathways which involve multiple appointments to initiate treatment present a barrier and increase the risk of loss to follow‐up [10], particularly among people experiencing homelessness or those not linked to a regular service. Approval of DBS by the Australian Therapeutic Goods Administration (government organisation responsible for the regulation of therapeutic goods) and other regulatory agencies as a standalone diagnostic tool could streamline pathways to care in the community by reducing the need for confirmatory testing and improving treatment initiation.

Few demographic or behavioural factors were associated with treatment initiation. While this pattern may indicate that DBS testing can support more equitable access to treatment, this interpretation should be considered in the context of the limited range of demographic and behavioural factors included in this analysis. However, Aboriginal and Torres Strait Islander participants testing in community settings had significantly lower odds of initiating treatment within 6 months. This finding identifies an important equity gap in the HCV care cascade and suggests that expanding access to testing alone may not be sufficient to achieve equitalbe treatment outcomes. Without targeted action, there is a risk that Aboriginal communities may be left behind in Australia's progress toward HCV elimination, further widening existing health inequities. Culturally safe, community‐led models of care developed in partnership with Aboriginal health services may help strengthen linkage to treatment and care [40]. Strategies such as peer support, incentives and integration with broader health services have been shown to improve engagement in care with all populations at risk of infection and should be considered for future programmes [41, 42, 43].

The findings of this study have important clinical and policy implications for the design of state and national strategies to achieve HCV elimination by 2030. The ongoing gap in HCV treatment initiation, particularly between people in community and prison settings and Aboriginal and non‐Aboriginal people, requires greater focus. While DBS testing has been shown to have overall acceptability among people at risk of HCV infection [5], the substantially lower treatment initiation observed in community compared with prison settings demonstrates that simplifying sample collection alone does not eliminate barriers in the cascade of care. The benefits of DBS scale‐up must be accompanied by targeted models of care to address barriers to treatment initiation and engagement in care, particularly in community contexts. Interventions shown to be effective include patient and provider education to improve HCV testing uptake, and integrated care, patient navigation, or care coordination models to strengthen linkage to treatment [44].

This study had several limitations. First, the NSW HIV and HCV DBS Pilot protocol was altered multiple times between 2017 and 2023 to expand eligibility criteria and reduce data collection burden for participants. As a result, certain variables were not collected consistently throughout the study, and people completing DBS registration prior to the protocol change in September 2019 were excluded. Additionally, the observed decline in HCV prevalence over time should be interpreted with caution. Changes in eligibility criteria may have influenced the characteristics of people accessing testing and, consequently, the prevalence estimates observed in different time periods. While the decline is consistent with broader trends in HCV prevalence following DAA scale‐up in Australia, changes in the composition of the tested population may also have contributed to these findings. Second, completeness of treatment initiation data varied across services due to different data collection processes, and the date at which participants were lost to follow‐up, or moved between services (including prison transfers) was not recorded. In addition, data regarding treatment initiation may have been more complete in prison settings, where participants typically received testing and treatment within the same health service. In contrast, participants tested in community settings were often referred to other providers for treatment, and treatment initiation occurring outside the reporting service may not have been captured. Consequently, treatment initiation in community settings may have been underestimated, which could have contributed to the observed difference in treatment uptake between community and prison settings. Third, models of care varied by site. While some sites were responsible for treatment initiation of participants, others without clinical capacity (including venepuncture for confirmatory testing) referred clients to alternative services for treatment workup. Treatment outcomes for participants referred to other providers were not routinely available and these participants were therefore excluded from analyses of treatment initiation. As referral practices were likely influenced by service characteristics and participant factors, exclusion of these participants may have introduced selection bias. Consequently, estimates of treatment initiation and factors associated with treatment uptake should be interpreted with caution. Finally, among participants who were released from prison before initiating treatment, treatment initiation data were unavailable, potentially leading to an underestimate of treatment initiation.

Overall, the prevalence of current HCV infection declined over time in community and prison settings, consistent with broader national trends following the scale‐up of DAA treatments. While treatment initiation was higher in prison (64% vs. 37% in community), the proportion initiating treatment has decreased in recent years, highlighting the need to strengthen treatment pathways. Across both settings, few factors were associated with treatment initiation, suggesting broadly equitable access based on the characteristics measured in this study. However, Aboriginal and/or Torres Strait Islander participants were less likely to initiate treatment in community settings, indicating a persistent gap in care. This study supports the integration of DBS into routine clinical care for HCV and reinforces the value of alternative testing strategies for people who experience barriers to traditional models of care. However, the marked difference in treatment initiation between community and prison settings demonstrates that expanding access to diagnosis alone is insufficient to achieve HCV elimination. While DBS can successfully increase access to testing at scale, testing scale‐up must be accompanied by strong, culturally safe, and integrated models of care that address barriers to treatment initiation and support engagement across the cascade of care.

## Author Contributions

Annabelle Stevens: formal analysis, investigation, resources, data curation, writing: original draft, writing: review and editing, visualisation, project administration. Anna Conway: formal analysis, investigation, data curation, writing: review and editing. Nigel Carrington: resources, data curation, project administration, writing: review and editing. Evan B. Cunningham: conceptualization, methodology, supervision, writing: review and editing. Alison D. Marshall: supervision, writing: review and editing. Janaki Amin: methodology, supervision, writing: review and editing. Phillip Read: investigation, resources, data curation, writing: review and editing. Tracey Brown: investigation, resources, writing: review and editing. Rick Varma: funding acquisition, project administration, supervision, writing: review and editing. Bianca Prain: funding acquisition, project administration, supervision, writing: review and editing. Philip Cunningham: funding acquisition, project administration, supervision, writing: review and editing. Gregory J. Dore: conceptualization, methodology, supervision, funding acquisition, writing: review and editing. Jason Grebely: conceptualization, methodology, supervision, funding acquisition, writing: review and editing.

## Funding

The work was supported by the NSW Ministry of Health. Staff at the NSW Ministry of Health had an active role in the design, conduct, and analysis of the study, interpretation of the data, and writing of the report. Some staff members were included as study co‐authors (Annabelle Stevens, Bianca Prain, Nigel Carrington).

## Conflicts of Interest

Jason Grebely is supported through National Health and Medical Research Council Investigator Grants (1 176 131, 2 036 888). The Kirby Institute is funded by the Australian Government Department of Health and Ageing. The views expressed in this publication do not necessarily represent the position of the Australian Government. This research was produced in whole or part by UNSW Sydney researchers and is subject to the UNSW Intellectual Property Policy. For the purposes of Open Access, the authors have applied a Creative Commons Attribution CC BY licence to any Author Accepted Manuscript (AAM) version arising from this submission.

## Data Availability

The data that support the findings of this study are available on request from the corresponding author. The data are not publicly available due to privacy or ethical restrictions.
